# A Differential Concentration-Dependent Effect of IVIg on Neutrophil Functions: Relevance for Anti-Microbial and Anti-Inflammatory Mechanisms

**DOI:** 10.1371/journal.pone.0026469

**Published:** 2011-10-31

**Authors:** Sarah Casulli, Selma Topçu, Lakhdar Fattoum, Stephan von Gunten, Hans-Uwe Simon, Jean-Luc Teillaud, Jagadeesh Bayry, Srini V. Kaveri, Carole Elbim

**Affiliations:** 1 Centre de Recherche des Cordeliers, Université Pierre et Marie Curie – Paris 6, UMR S 872, Paris, F-75006 France and Université Paris Descartes, UMR S 872, Paris, F-75006 France; INSERM, U872, Paris, F-75006, France; 2 Institute of Pharmacology, University of Bern, Bern, Switzerland; The Scripps Research Institute, United States of America

## Abstract

**Background:**

Polymorphonuclear neutrophils (PMN) play a key role in host defences against invading microorganisms but can also potentiate detrimental inflammatory reactions in case of excessive or misdirected responses. Intravenous immunoglobulins (IVIg) are used to treat patients with immune deficiencies and, at higher doses, in autoimmune, allergic and systemic inflammatory disorders.

**Methodology/Principal Findings:**

We used flow cytometry to examine the effects of IVIg on PMN functions and survival, using whole-blood conditions in order to avoid artifacts due to isolation procedures. IVIg at low concentrations induced PMN activation, as reflected by decreased L-selectin and increased CD11b expression at the PMN surface, oxidative burst enhancement, and prolonged cell survival. In contrast, IVIg at higher concentrations inhibited LPS-induced CD11b degranulation and oxidative burst priming, and counteracted LPS-induced PMN lifespan prolongation.

**Conclusions/Significance:**

IVIg appears to have differential, concentration-dependent effects on PMN, possibly supporting the use of IVIg as either an anti-microbial or an anti-inflammatory agent.

## Introduction

Intravenous immunoglobulin (IVIg) is a therapeutic preparation of normal human polyclonal IgG derived from pooled plasma from a large number of healthy donors. Initially used as replacement therapy for patients with primary and secondary immune deficiencies, IVIg is now also widely used for the treatment of a variety of autoimmune, allergic and systemic inflammatory disorders, at high immunomodulatory doses [1], [2], [3], [4], [5], [6], [7]. The ability of exogenous IVIg to prevent infections in immunodeficient patients has been attributed to neutralization of pathogens and bacterial toxins by specific antibodies. High-dose IVIg is known to dampen the activity of dendritic cells, lymphocytes, endothelial cells, monocytes and macrophages, and to modulate cytokine synthesis and the complement system [8], [9], [10], [11]. However, a more precise understanding of the mechanisms by which IVIg exerts its therapeutic effects is needed for more rational use of this drug.

Polymorphonuclear neutrophils (PMN) are key components of the first line of defense against invading microorganisms. In response to pathogens, blood PMN rapidly migrate to inflamed tissues, where their activation triggers microbicidal mechanisms such as release of proteolytic enzymes and antimicrobial peptides, and rapid production of reactive oxygen species (ROS), in the so-called oxidative burst. This process is critical for bacterial killing but can also cause severe bystander tissue injury if excessive or inappropriate [12]. After bacterial killing, PMN die by apoptosis and are then recognized and phagocytozed by macrophages [13]. PMN are usually short-lived immune cells, but prolongation of their life span is critical for their tissue accumulation and pathogen destruction [14]. Inappropriate PMN survival and persistence at sites of inflammation can lead to the release of cytotoxic contents into the extracellular environment, which may contribute to the pathophysiology of chronic inflammatory and allergic diseases [15], [16], [17].

The few published studies on the effects of IVIg on PMN yielded conflicting results. Some authors report that IVIg alone triggers PMN degranulation [18] while others describe an increase in apoptotic PMN [19], which exhibit lower pro-inflammatory activity [20]. It has been also suggested that IVIg may act as an immunomodulator, inhibiting stimulus-induced PMN degranulation [21] and survival [22]. However, Jarius et al reported a priming effect of IVIg on the PMN oxidative burst in response to TNF [23]. Importantly, most of these studies used PMN isolated from their blood environment by various procedures that may differently modulate cell responses [24], [25].

The aim of this study was to re-examine the effect of IVIg on key PMN functional characteristics, including adhesion molecule expression, ROS production and survival, using resting and LPS-stimulated PMN in whole-blood conditions in order to minimize activation due to isolation procedures, an approach that we have previously validated [26], [27].

## Results

### IVIg at low concentrations enhances PMN activation

**Figure 1:**
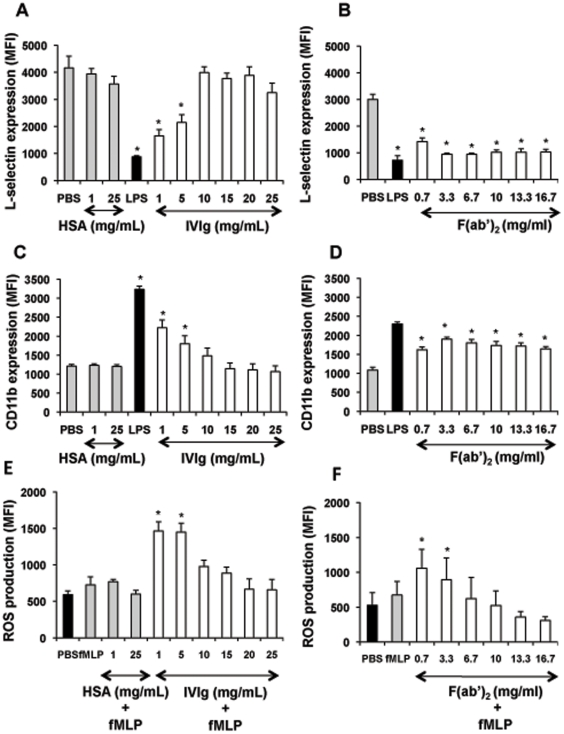
IVIg at low concentrations activates PMN functions. Panels A, B, C and D: Effect of IVIg and F(ab′)_2_ fragments on adhesion molecule expression at the PMN surface. Whole-blood samples (500 µl) were incubated in a water bath with gentle horizontal agitation at 37°C for 45 min with PBS, HSA (1 or 25 mg/ml), or IVIg (1–25 mg/ml) (panel A, C) or equimolar concentrations of F(ab′)_2_ fragments (panel B, D). LPS (10 ng/ml, 45 min) was used as a positive control. Samples were then stained with PE-anti-human CD11b or anti-L-selectin at 4°C for 30 min. Samples stained with anti-L-selectin were washed with ice-cold PBS and incubated at 4°C for 30 min with FITC-goat anti-mouse. Then, erythrocytes were lysed with FACS lysing solution and white blood cells were resuspended in 1% paraformaldehyde-PBS and analysed by flow cytometry. Nonspecific Ab binding was determined on cells incubated with the same concentrations of an irrelevant Ab of the same isotype. Results are expressed as mean fluorescence intensity (MFI). Values are means ± sem (n = 6). ^*^ Significantly different from sample incubated with PBS (p<0.05). Panels E and F: Effect of IVIg and F(ab′)_2_ fragments on the PMN oxidative burst in response to formyl peptides. Whole-blood samples (500 µl) were pre-treated with hydroethidine (1500 ng/ml) for 15 min in a water bath with gentle horizontal agitation at 37°C. The samples were then incubated at 37°C for 45 min with PBS, HSA (1 or 25 mg/ml), or IVIg (1–25 mg/ml) (panel E) or equimolar concentrations of F(ab′)_2_ (panel F) before stimulation with fMLP (10^−6^ M) for 5 min. A sample incubated with LPS (10 ng/ml, 45 min) then with fMLP (10^−6^ M, 5 min) was used as a positive control. Erythrocytes were lysed with FACS lysing solution and white blood cells were resuspended in 1% paraformaldehyde-PBS. Samples were analysed by flow cytometry. Results are expressed as mean fluorescence intensity (MFI). Values are means ± sem (n = 3). * Significantly different from sample incubated with fMLP (p<0.05).

One of the main steps in PMN migration from the bloodstream to an inflammatory site is the modulation of adhesion molecule expression on both PMN and endothelial cells. In particular, stimulus-induced shedding of L-selectin (CD62L), followed by increased expression of the β2 integrin CD11b/CD18, is a major mechanism underlying transendothelial migration [28]. IVIg is used at two distinct dose regimens in clinical practice: in primary and secondary immune deficiencies, IVIg is administered as a substitutive agent at 400–500 mg/kg body weight whereas in autoimmune and inflammatory conditions, IVIg is given at 1 g/kg body weight. Accordingly, in the *in vitro* studies, these doses correspond to 1–5 mg/ml or 25 mg/ml [29]. As shown in Figure 1A and C, treatment of whole blood with IVIg at low concentrations (1–5 mg/ml) reduced CD62L expression and enhanced CD11b expression at the PMN surface, as compared to the PBS control.

We have previously reported that, in whole blood, a single stimulus gives rise to minimal ROS production by PMN [30]. We therefore studied the effect of IVIg on the oxidative burst in response to bacterial formyl peptides (fMLP). Pretreatment of whole blood with IVIg at low concentrations followed by fMLP stimulation significantly increased ROS production as compared to fMLP alone (Figure 1E).

Similar results were obtained with equimolar amounts of F(ab′)_2_ fragments derived from IVIg (Fig. 1B, D, F), whereas no effect was observed with equimolar amounts of Fc fragments (not shown).

HSA, used as an irrelevant protein, did not exert any significant effect on the expression of PMN adhesion molecules and ROS production at 1 and 25 mg/ml (Figure 1A, 1C, 1E) as well as at all the other doses tested (5, 10, 15 and 20 mg/ml) (not shown).

### IVIg at low concentrations delays PMN apoptosis

**Figure 2:**
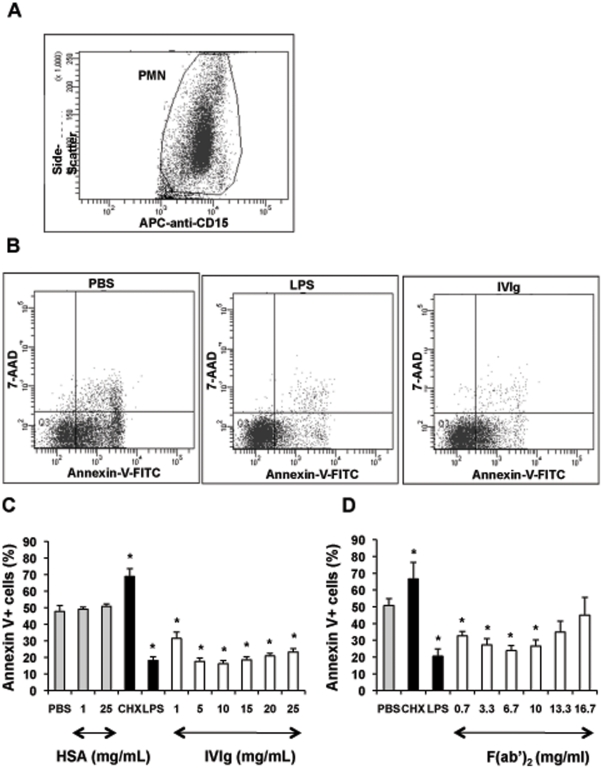
IVIg at low concentrations delays PMN apoptosis. Whole-blood samples (500 µl) were incubated in 24-well tissue culture plates at 37°C for 20 h with 5% CO_2_ with PBS, HSA (1 or 25 mg/ml), or IVIg (1–25 mg/ml) (Panel C) or equimolar concentrations of F(ab′)_2_ (Panel D). Cycloheximide (CHX) (10 µg/ml) and LPS (10 ng/ml) were used as proapoptotic and antiapoptotic controls, respectively. Samples (100 µl) were then washed twice in PBS, incubated at 4°C with APC-anti-CD15 for 15 min, and stained with FITC-annexin V for 15 min. After dilution in PBS (500 µl), the samples were incubated with 7-AAD for 15 minutes at room temperature and analysed immediately by flow cytometry as described in Materials and Methods. Anti-CD15 fluorescence was used to identify PMN as CD15^+^ cells and to gate out other cells, erythrocytes, and debris. A gate was drawn around the PMN population (Panel A). Fluorescence analysis was performed on this gate (Panel B). The combination of FITC-annexin V and 7-AAD was used to distinguish early apoptotic cells (annexin V^+^/7-AAD^−^), from late apoptotic cells (annexin V^+^/7-AAD^+^), necrotic cells (annexin V^−^/7-AAD^+^) and viable cells (unstained) after incubation with PBS, CHX, LPS or IVIg (1–25 mg/ml). Results are expressed as the percentage of total apoptotic cells (early and late apoptotic cells). The proportion of necrotic cells was always below 2%. Values are means ± sem (n = 3). * Significantly different from sample incubated with PBS (p<0.05).

Bloodstream PMN have a short half-life, dying mainly by apoptosis [31]. Prolongation of their lifespan is critical for efficient pathogen destruction. We therefore investigated the effect of IVIg on PMN apoptosis. Whole blood PMN cultured at 37°C died rapidly by apoptosis, about 50% of cells being annexin V^+^ after 20 h. As previously reported [26], apoptosis was accelerated by cycloheximide (10 µg/ml) and delayed by LPS (10 ng/ml). The percentage of apoptotic cells in whole-blood samples incubated with intact IVIg or F(ab′)_2_ fragments was significantly lower than in the PBS control (Figure 2C and D). This anti-apoptotic effect appears as soon as 8 hours and was not observed with IVIg-derived Fc fragments (not shown). HSA did not exert any significant effect on PMN apoptosis at 1 and 25 mg/ml (Figure 2C) as well as at all the other doses tested (5, 10, 15 and 20 mg/ml) (not shown).

### IVIg counteracts LPS-induced PMN activation and survival

**Figure 3:**
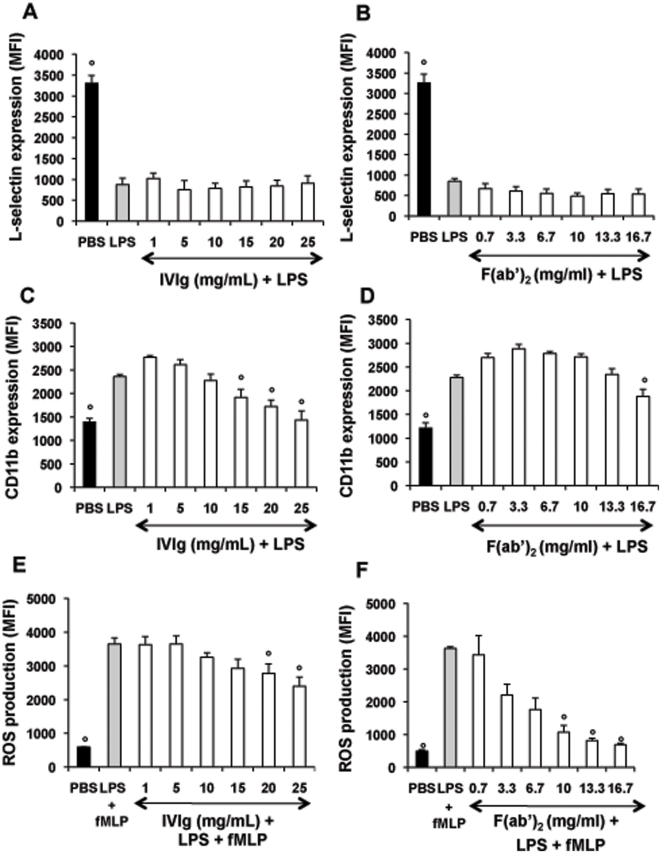
IVIg at high concentrations inhibits PMN responses to LPS stimulation. Panels A, B, C and D: Effect of IVIg and F(ab′)2 fragments on LPS modulation of adhesion molecule expression at the PMN surface. Whole-blood samples were incubated at 37°C for 15 min with PBS, IVIg (1–25 mg/ml) (panel A, C) or equimolar concentrations of F(ab′)_2_ (panel B, D) and then with LPS (10 ng/ml) for 30 min. Samples were then stained with PE-anti-human CD11b or anti-L-selectin as described in the legend of Figure 1. Results are expressed as mean fluorescence intensity (MFI). Values are means ± sem (n = 3). ° Significantly different from sample incubated with LPS (p<0.05). Panels E and F: Effect of IVIg and F(ab′)_2_ fragments on LPS priming of the PMN oxidative burst in response to formyl peptides. Whole-blood samples (500 µl) were pre-treated with hydroethidine HE and incubated at 37°C for 15 min with PBS, IVIg (1–25 mg/ml) (panel E) or equimolar concentrations of F(ab′)_2_ (panel F). Samples were then incubated with LPS and fMLP as described in the legend of Figure 1. Results are expressed as mean fluorescence intensity (MFI). Values are means ± sem (n = 3). * Significantly different from sample incubated with LPS+fMLP (p<0.05).

**Figure 4:**
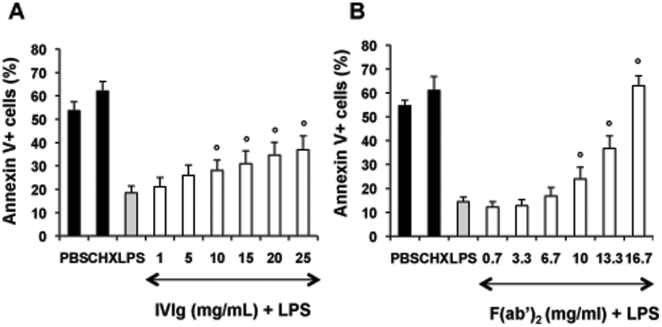
IVIg at high concentrations inhibits the LPS-induced prolongation of PMN survival. Whole-blood samples (500 µl) were incubated in 24-well tissue culture plates at 37°C with 5% CO_2_ with PBS, IVIg (1–25 mg/ml) (panel A) or equimolar concentrations of F(ab′)_2_ (panel B) for 15 min and then with LPS (10 ng/ml) for 20 h. Samples were then stained with annexin V and 7-AAD as described in the legend of Figure 1. Results are expressed as the percentage of total apoptotic cells (early and late apoptotic cells). Values are means ± sem (n = 3). * Significantly different from sample incubated with LPS (p<0.05).

We then explored the capacity of IVIg to modulate PMN responses to LPS, used as a pro-inflammatory stimulus. Both CD11b expression at the PMN surface and ROS production were significantly lower in samples incubated with LPS+IVIg at high concentrations (15–25 mg/ml) than in samples incubated with LPS alone (Figure 3C and E). In addition, IVIg significantly inhibited LPS-induced PMN survival (Figure 4A). Similar results on PMN lifespan were also obtained with other TLR ligands such as TLR1/2 or TLR7/8 ligands (not shown). Fc fragments had no significant effect on LPS-treated PMN (not shown), while F(ab′)_2_ fragments countered LPS-induced modulations (Figure 3D and F, and Figure 4B) more efficiently than IVIg. In particular, F(ab′)_2_ fragments had a pro-apoptotic effect on LPS-stimulated PMN.

To determine whether this effect was due to the presence of auto-antibodies to sialic acid-binding Ig-like lectin 9 (Siglec-9) in the IVIg preparation [19], [32], we tested the effect of anti-Siglec-9-depleted IVIg on LPS-induced PMN survival. In keeping with previous data obtained with isolated PMN treated with GM-CSF [19], [33], we found that the death-inducing effect of IVIg in the presence of LPS was totally abolished when anti-Siglec-9-depleted IVIg was used (not shown).

## Discussion

It is important to note that we analyzed PMN functions and apoptosis by flow cytometry in whole-blood conditions, whereas previous studies used isolated PMN. Indeed, PMN isolation procedures have been reported to alter PMN responses [24], [25], [26]. In addition, interactions between cellular elements have been reported to be important for maintaining PMN viability [34].

Our results show that low concentrations of IVIg, as well as F(ab′)_2_ fragments, induce PMN activation, as reflected by decreased L-selectin expression, increased CD11b/CD18 adhesion molecule expression, and enhanced ROS production. These effects may be related to ANCA-like antibodies reported to be present in IVIg preparations [23]. Further, it is possible that IVIG at higher concentrations trigger inhibitory signalling pathways as has been demonstrated in the case of dendritic cells, B and T lymphocytes [9]. Using whole-blood PMN, we found that IVIg-induced PMN activation is also associated with prolonged PMN survival. This anti-apoptotic effect may be due to natural anti-Fas antibodies directed against the CH11 epitope leading to an antagonist effect, although it has been shown that IVIg contains both pro-apoptotic and anti-apoptotic anti-Fas antibodies [35]. On the contrary, it has been previously reported that IVIg alone induces PMN apoptosis [19], [33]. These discrepancies may be related to differences in antagonist and agonists anti-Fas activities reported in various IVIg preparations [36]. While this anti-apoptotic effect was not observed with Fc fragments, the involvement of FcγRII and FcγRIII cannot be ruled out.

Immunoglobulin replacement therapy significantly reduces the incidence of lower airway infections and pneumonia in patients with primary immunodeficiencies [37]. Although IVIg is known to contain antibodies against various pathogens [38], [39], its beneficial effects might not be due solely to pathogen neutralization. Natural antibodies present in IVIg enhance opsonization and thus promote phagocytosis and antibody-mediated cytotoxicity [40]. Neutrophils are the first cells to be recruited to sites of infection in response to a variety of inflammatory stimuli, and protect the host by engulfing, killing and digesting infectious agents. The PMN activation and lifespan prolongation observed here with low concentrations of IVIg could enhance defenses against microbial pathogens *in vivo*. In addition, inhibition of PMN apoptosis by IVIg may contribute to reducing the incidence and severity of neutropenia, which is a feature of several antibody deficiencies [41].

Contrary to low concentrations, high concentrations of IVIg inhibited the LPS-induced increase in CD11b expression and ROS production by PMN, as well as the LPS-induced delay in PMN apoptosis. In keeping with previous data obtained with isolated PMN treated with GM-CSF [19] [33], PMN apoptosis was not delayed by anti-Siglec-9 depleted IVIg. This result emphasizes the hypothesis that anti-Siglec-9 Abs contained in IVIg preparation exert a pro-apoptotic effect under certain inflammatory conditions that sensitize PMN towards the induction of apoptosis [42] [43]. Siglec receptors belong to the Ig supergene family and are characterized by the presence of an N-terminal V-set domain that binds sialic acid [44]. These receptors are predominantly expressed on cells of the innate and adaptative immune system, in a cell-type-restricted and differentiation-dependent manner [44]. For instance, Siglec-9 is predominantly expressed on monocytes and PMN [45]. It has been clearly established that Siglec-9 ligation triggers PMN apoptosis through caspase-dependent and -independent pathways [19] [46]. Engagement of Siglec-9 has also been reported to dampen neutrophil responses, including the oxidative burst [47]. Finally, neutralization of LPS by anti-LPS antibodies within IVIg preparations cannot be ruled out. Presence of anti-LPS antibodies in IVIg has been previously documented [48]. However, we have also shown that IVIg could block human dendritic cell maturation when cells are pre-exposed to LPS followed by treatment with IVIg [48]. Therefore, in addition to direct neutralization of LPS, other mechanisms of counteracting its effect on PMN activation and survival (such as blocking the interaction of LPS with TLR-4 and associated molecules, and inhibiting down-stream signaling) might be implicated in IVIg-mediated regulation of PMN functions. Furthermore, the pro-apoptotic effect of IVIg on whole-blood PMN may also be related to IVIg-induced down-modulation of anti-apoptotic cytokines by activated monocytes [9].

The lifespan of PMN increases significantly when these cells migrate out of the circulation and into sites of inflammation, where they encounter various pro-inflammatory mediators. Inappropriate PMN survival and persistence at sites of inflammation and/or excessive PMN activation lead to the release of cytotoxic contents into the extracellular environment, a process that contributes to many inflammatory disorders [49], [50], [51]. The ability of high concentrations of IVIg to dampen PMN responses to pro-inflammatory stimuli may thus contribute to the beneficial effect of IVIg observed in autoimmune and inflammatory diseases, adding to the long list of its non exclusive mechanisms of action [8] [9]. Finally, the ability of IVIg to counteract the LPS-induced delay in PMN apoptosis supports the use of IVIg in allergic patients, and especially in asthma. In some asthma patients, in whom the inflammation is non-atopic, non-IgE-dependent and non-eosinophilic, it has been reported that airway neutrophilia, related to delayed neutrophil apoptosis [16], correlates with clinical severity [52]. These forms of asthma respond poorly to corticosteroid therapy.

In conclusion, this study points to a differential effect of IVIg on PMN, depending both on the IVIg concentration and the pro-inflammatory environment. Low concentrations of IVIg induce PMN activation and prolongation of lifespan, while high concentrations counteract LPS-induced PMN activation. These findings support the use of IVIg either as an antimicrobial or an anti-inflammatory agent.

## Materials and Methods

### Therapeutic intravenous immunoglobulin

A therapeutic IVIg preparation (Sandoglobulin®) was used for all experiments, after extensive dialysis. F(ab′)_2_ fragments were prepared from IVIg by pepsin digestion (2% wt/wt; Sigma, St Louis MO) followed by chromatography on a protein A Sepharose column (Pharmacia, Uppsala, Sweden). F(ab′)_2_ fragments were shown to be free of intact IgG and Fc fragments by means of SDS-PAGE and ELISA (not shown). Fc fragments were obtained from Dr. M.-C. Bonnet, Pasteur Mérieux Sérums & Vaccins, Marcy L'Etoile, France (Debre, 1993 #440). Anti-Siglec-9 antibodies (Abs) were depleted from IVIg preparations by affinity chromatography, as reported in [19].

### Incubation of whole blood with IVIg

Fresh whole-blood samples were obtained, after written consent, from healthy donors purchased from Hôpital Hôtel Dieu, Etablissement Français du Sang (EFS), Paris, France, after ethical approval for the use of such material by the INSERM's and EFS's ethic committees: convention 09/EFS/024. Samples were treated at 37°C with PBS, HSA (1–25 mg/ml), IVIg (1–25 mg/ml) or equimolar concentrations of F(ab′)_2_ or Fc fragments for various periods of time. In some experiments, blood samples were first incubated with IVIg or F(ab′)_2_ fragments and then stimulated with LPS from *E. coli* serotype R515 (LPS, 10 ng/ml; Invivogen, San Diego, CA).

### Determination of adhesion molecule expression at the PMN surface

After incubation with PBS, HSA or IVIg as described above, samples were then stained at 4°C for 30 min with PE-anti-human CD11b (Dakopatts, Glostrup, Denmark) or purified anti-L-selectin (BD Biosciences, San Jose, CA). To study L-selectin expression, samples were washed with ice-cold PBS and incubated at 4°C for 30 min with FITC-goat anti-mouse (Ab Nordic Immunology, Tilburg, The Netherlands). Erythrocytes were lysed with FACS lysing solution (BD Biosciences) and white blood cells were resuspended in 1% paraformaldehyde–PBS. Nonspecific Ab binding was determined on cells incubated with the same concentration of an irrelevant Ab of the same isotype.

### Measurement of the PMN oxidative burst

Superoxide anion (O_2_^−°^) production was measured with a flow cytometric assay derived from the hydroethidine (HE) oxidation technique [26], [53]. Whole-blood samples (500 µl) were loaded for 15 min with 1500 ng/ml HE (Fluka, Buchs, Switzerland) at 37°C and then incubated with PBS, HSA or IVIg as described above. Samples were then treated with PBS or 10^−6^ M fMLP (Sigma Chemical CO., St Louis, MO) for 5 min. Red cells were lysed as described above and white cells were resuspended in 1% paraformaldehyde-PBS.

### Measurement of PMN apoptosis

Apoptosis of PMN in whole blood was quantified by using annexin V and the impermeant nuclear dye 7-amino-actinomycin D (7-AAD) as previously described [26], [54]. PMN apoptosis was measured immediately after sampling or after incubation in 24-well tissue culture plates at 37°C with PBS, HSA or IVIg as described above. Cycloheximide (Calbiochem, La Jolla, CA) (10 µg/ml) and LPS (10 ng/ml) were used as pro-apoptotic and anti-apoptotic controls, respectively [26]. Whole-blood samples (100 µl) were then washed twice in PBS, incubated with APC-anti-CD15 for 15 min, and then incubated with APC-annexin V for 15 minutes. After dilution in PBS (500 µl), the samples were incubated with 7-AAD (BD Biosciences) at room temperature for 15 minutes and analyzed immediately by flow cytometry.

### Flow cytometry

A Becton Dickinson LSRII flow cytometer was used. PMN were analyzed with DIVA software. To measure apoptosis in whole blood, PMN were identified as CD15^high^ cells and 2×10^5^ events were counted per sample. A gate was drawn around the PMN population (see Figure 2 Panel A). Fluorescence analysis was performed on this gate (see Figure 2 Panel B). The combination of FITC-annexin V and 7-AAD was used to distinguish early apoptotic cells (annexin V^+^/7-AAD^−^), from late apoptotic cells (annexin V^+^/7-AAD^+^), necrotic cells (annexin V^−^/7-AAD^+^) and viable cells (unstained). In other experiments, forward and side scatter were used to identify the PMN population and to gate out other cells and debris; 10 000 events were counted per sample.

### Statistical analysis

Data are reported as means ± SEM. Comparisons were based on ANOVA and Tukey's Post Hoc tests, using Prism 3.0 software (Graph Pad software). Significance was assumed at p<0.05.
